# Southern Ocean carbon export efficiency in relation to temperature and primary productivity

**DOI:** 10.1038/s41598-020-70417-z

**Published:** 2020-08-10

**Authors:** Gaojing Fan, Zhengbing Han, Wentao Ma, Shuangling Chen, Fei Chai, Matthew R. Mazloff, Jianming Pan, Haisheng Zhang

**Affiliations:** 1 College of Marine Science and Technology, China University of Geosciences, Wuhan, 430074 China; 2 Key Laboratory of Marine Ecosystem Dynamics, Second Institute of Oceanography, Ministry of Natural Resources, Hangzhou, 310012 China; 3 State Key Laboratory of Satellite Ocean Environment Dynamics, Second Institute of Oceanography, Ministry of Natural Resources, Hangzhou, 310012 China; 4 School of Marine Sciences, University of Maine, Orono, ME 04469 USA; 5 Scripps Institution of Oceanography, University of California San Diego, La Jolla, CA 92093 USA

**Keywords:** Carbon cycle, Carbon cycle, Carbon cycle, Carbon cycle, Marine biology

## Abstract

Satellite remote sensing and numerical models are widely used to estimate large-scale variations in ocean carbon export, but the relationship between export efficiency (e-ratio) of sinking organic carbon out of the surface ocean and its drivers remains poorly understood, especially in the Southern Ocean. Here, we assess the effects of temperature and primary productivity on e-ratio by combining particulate organic carbon export flux from in situ measurements during 1997–2013, environmental parameters from satellite products, and outputs from ocean biogeochemical models in the Southern Ocean. Results show that “High Productivity Low E-ratio” (HPLE) is a common phenomenon in the Subantarctic Zone and the Polar Frontal Zone, but not the Antarctic Zone. The e-ratio shows little dependence on temperature below 6 °C. Our results support the hypothesis that the HPLE phenomenon is due to the large contribution of non-sinking organic carbon. Both temperature and ballast minerals play less important roles in controlling e-ratio than ecosystem structure at low temperatures. These findings suggest that non-sinking organic carbon, ecosystem structure, and region-specific parameterizations of e-ratio are key factors to quantify the carbon export in the Southern Ocean.

## Introduction

Characterized by unique oceanographic features, such as the convergent zone and upwelling of nutrient-rich deep waters, the Southern Ocean (SO) plays a dominant role in global ocean carbon uptake and in nutrient supply to low latitudes^1,2^. The strength and efficiency of the biological carbon pump (BCP) that transfers organic carbon from surface to deep waters strongly influences the degree of carbon sequestration^3^, and subsequently regulates long-term atmospheric CO_2_ levels and global climate^4,5^. Earth system models indicate that under a high CO_2_ emission scenario the increase of carbon export in the SO will initiate nutrient trapping and accompany the decrease in fisheries yields by over 20% globally and by 60% in the North Atlantic due to the decline of nutrient supply and hence primary productivity in the euphotic zone^6^. Therefore, an accurate estimate of the BCP magnitude in the SO is important for predicting the responses of global climate and marine ecology.

Export production (EP), an important component of the BCP, represents the fraction of particulate organic carbon (POC) produced by phytoplankton exported out of the upper ocean (the euphotic zone, mixed layer, or 100 m based on different definitions of export depth horizon). A variety of empirical or mechanistic models have been developed to extrapolate the sparse field observations of EP to larger temporal and spatial scales^7–14^. These models are largely based on mathematical relationships between export efficiency (e-ratio), net primary productivity (NPP), and sea surface temperature (SST). Here, e-ratio refers to the ratio between EP and NPP. It was found that e-ratio is positively correlated with NPP and negatively correlated with SST in the global ocean by compiling existing observations of carbon export flux and NPP^7–11^. However, Maiti et al.^12^ found that e-ratio shows a negative relationship with NPP and a weak relationship with SST at latitudes south of 40° S based on 140 field observations of NPP and EP. These contradictory findings would result in large biases or errors in the estimate of carbon export production and efficiency in the SO.

Several field studies revealed the presence of a High Productivity Low E-ratio (HPLE) pattern in the SO^15–19^. A global biogeochemical model also reproduced this pattern when SST was < 7 °C^20^. Various hypotheses have been proposed to explain the HPLE pattern, including the time lag between export and production^20–22^, bacteria activity^23^, dissolved organic carbon (DOC) export^12^, and zooplankton regulation via grazing and excretion^24,25^. Besides, some studies questioned whether the HPLE pattern is just a statistical artifact when examining the relationship between the NPP-dependent e-ratio and NPP itself^26,27^. As for the weak relationship between e-ratio and SST, after reanalyzing the data compilation of Maiti et al.^12^ and silicate concentration fields from the World Ocean Atlas 2013, Britten et al.^14^ proposed “temperature-ballast hypothesis”, which suggests that the effect of temperature on e-ratio is masked by variations in silica-ballast. Yet, Le Moigne et al.^28^ suggested that ecosystem functions (e.g., grazing intensity via packaging of slowly sinking phytoplankton cells into large fecal pellets) are potentially more important drivers than ballast minerals in facilitating efficient export of POC in the SO, despite the ~ 40% contribution of mineral ballast (mostly biogenic silica) on the total POC export. Other studies noted that POC flux determines the flux of mineral ballast materials to depth, rather than the other way around^29,30^. Nevertheless, the relationships between e-ratio, NPP, and SST in the SO remain unclear.

**Figure 1:**
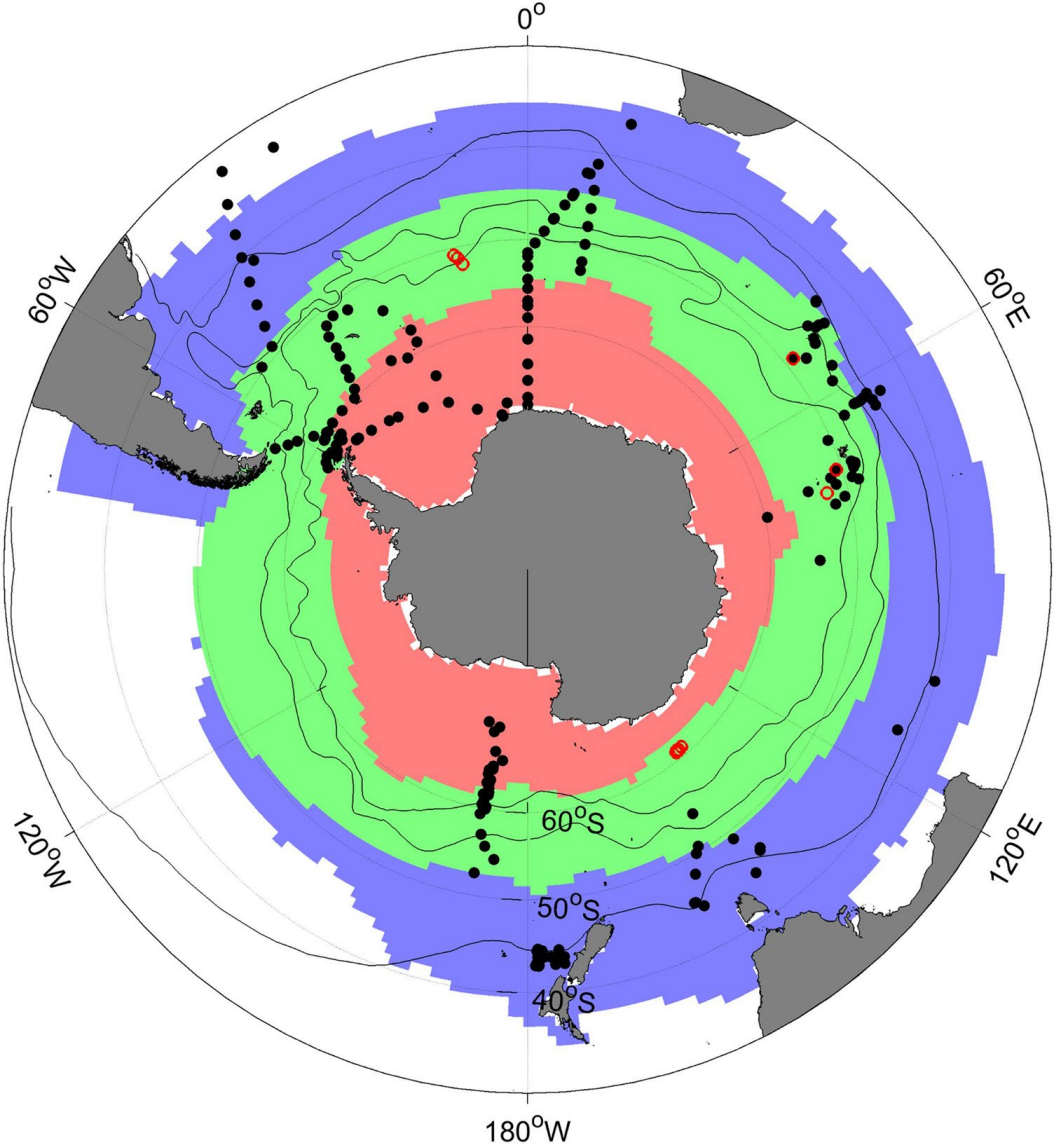
Map of the Southern Ocean with the locations of sampling stations overlaid. The POC export data at 100 ± 10 m was determined based on ^234^Th-method (black dot, N = 218) and drifting sediment trap (red circle, N = 13). Different background colors represent the three biomes defined by Fay and McKinley^31^: the Subantarctic Zone (SAZ, blue), the Polar Frontal Zone (PFZ, green), and the Antarctic Zone (AZ, red). Three sectors are defined: the Pacific sector (120° E–65° W), the Indian sector (20° E–120° E), and the Atlantic sector (65° W–20° E). The black contours from south to north represent the mean positions of the Antarctic Polar Front (APF), Subantarctic Front (SAF), and Subtropical Front (STF), respectively.

In this paper, we revisit the relationships between export efficiency, NPP, and temperature using a comprehensive dataset of POC export flux measurements compiled from published literature, satellite-derived SST and NPP products, and model outputs to answer the following questions: (1) Is e-ratio independent of temperature in the SO? (2) Is the HPLE pattern widespread in the SO over different spatial and temporal scales? And (3) what are the potential controls on the responses of e-ratio to NPP and SST? To examine the regional and temporal variation in the relationships between e-ratio, NPP, and SST, we partitioned the SO into different biomes and sectors, and also studied seasonality (Fig. 1). Three mean biomes, the Subantarctic Zone (SAZ), Polar Frontal Zone (PFZ), and Antarctic Zone (AZ) of the SO, were used from Fay and McKinley^31^ who combined the climatological SST, chlorophyll-a concentration, sea-ice fraction, and maximum mixed layer depth to define global open-ocean biomes and they could capture the biogeochemical functioning at the basin scale. Based on the locations of EP measurements, we defined three sectors: the Pacific sector (120° E–65° W), the Indian sector (20°–120° E), and the Atlantic sector (65° W–20° E). We also attempted to explore the seasonal variability of the relationships in the austral spring (September, October, and November), summer (December, January, and February), and autumn (March, April, and May). We then utilized outputs from both global and regional coupled physical-biogeochemical models to investigate the possible causes for the identified patterns and relationships between e-ratio, NPP, and SST.

## Results and discussion

### Correlations between e-ratio, NPP, and SST

**Figure 2:**
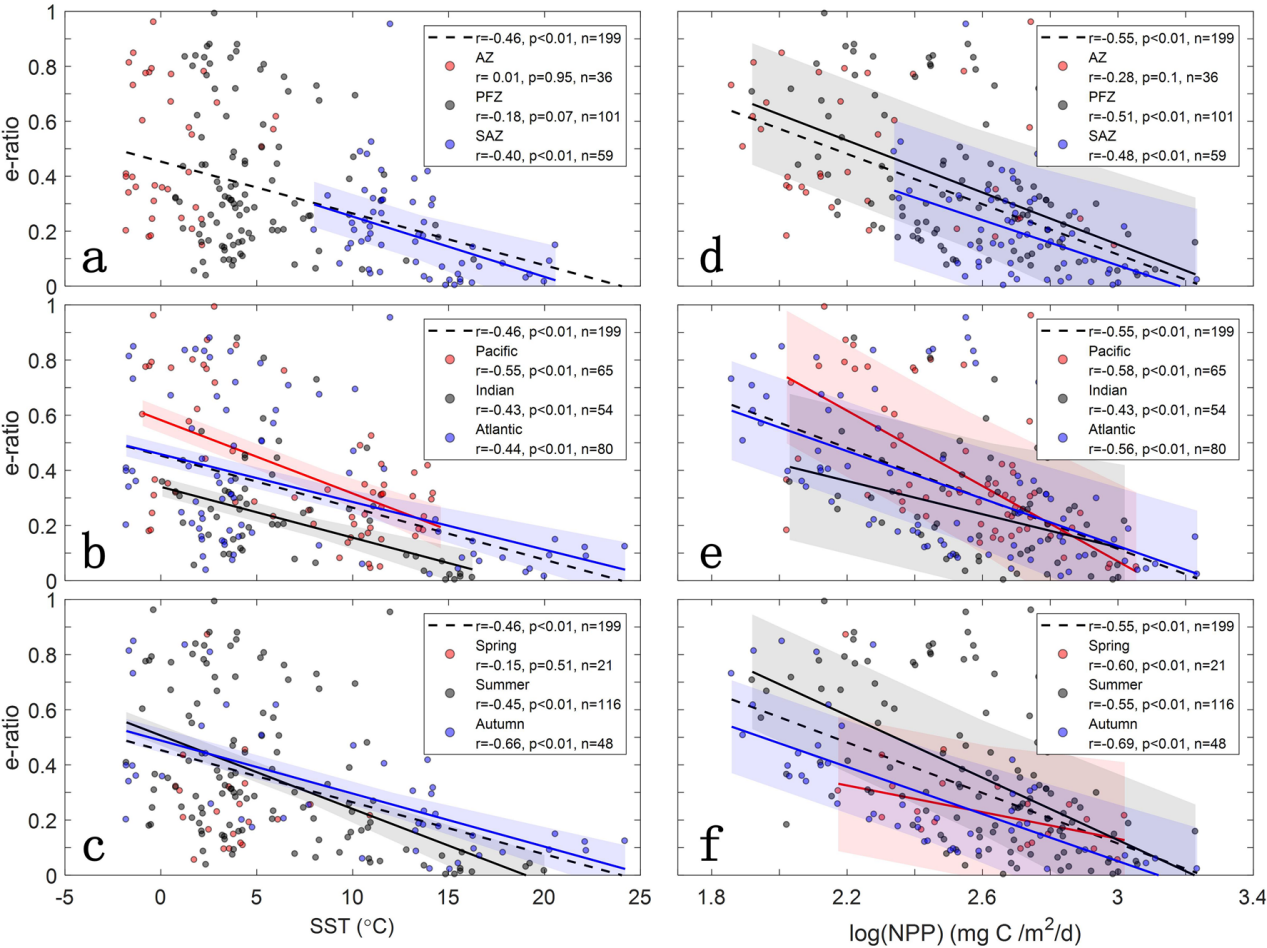
Relationship between e-ratio and SST **(a–c)**, and between e-ratio and NPP **(d–f)** in different subregions, including biomes **(a, d)**, sectors **(b, e)**, and seasons **(c, f)**. The dotted line in each panel represents the linear correlation for all stations, while three solid lines plot the significant linear correlations (*p* < 0.05) for each subregion or season (red: AZ, Pacific Ocean, and spring; black: PFZ, Indian Ocean, and summer; blue: SAZ, Atlantic Ocean, and autumn). The shaded areas represent the standard error around the fitted solid lines. Pearson’s correlation coefficients (*r*-value) and *p*-values are shown. Note that all e-ratio (EP/NPP) values are calculated using VGPM NPP.

According to the definition, the e-ratio estimate is affected by the uncertainty in satellite-based NPP inferences. Therefore, we compared different satellite-based NPP products (VGPM, Eppley-VGPM, and CbPM) and e-ratio values with in situ estimates. The e-ratio values derived from these NPP products have no significant difference with in situ estimates (see “Methods” for details) and all yielded similar correlation patterns with SST or NPP, relatively insensitive to satellite NPP algorithms (Fig. 2 and Supplementary Figs. S1-S2). Figure 2 shows the relationships between e-ratio, SST, and NPP in six subregions and three seasons. The Pearson’s correlation coefficient (*r-*value) between e-ratio and SST and the associated significance level (*p*-value) indicate that e-ratio decreases with temperature over the entire Southern Ocean (*r* = − 0.66 to − 0.4, *p* < 0.01; Fig. 2a–c), consistent with previous studies that suggested the rate of respiration increases faster than photosynthesis with rising temperature, leading to a decrease in carbon export efficiency^32,33^. Exceptions to this relationship are found in the AZ and PFZ; both exhibit insignificant correlations between e-ratio and SST (*p* > 0.05). We also found that the e-ratio was uncorrelated to SST in austral spring, whereas only 21 EP measurements were collected in this season and they were all in the PFZ (Fig. 2c). Thus the lack of correlation is likely due to a sampling bias. Our finding contradicts the traditional view of higher e-ratio in colder waters, but agrees with the results reported by Maiti et al.^12^, which found no negative relationship between export efficiency and temperature in the SO.

Figure 2d–f shows that e-ratio has a clear negative correlation with NPP in most regions and seasons (*r* = − 0.69 to − 0.43, *p* < 0.01), which is independent on biome, sector, or season. This result confirms the previous finding of the relationship between e-ratio and NPP^12^. However, in the AZ there is no statistically significant correlation above the 95% confidence level (*r* = − 0.28, *p* > 0.05; Fig. 2d) although the relationship between e-ratio and NPP is generally negative, and this challenges the current prevailing viewpoint that POC export is closely related to NPP^8,11^. The independence of the relationship between e-ratio and NPP on satellite NPP algorithms was further examined by testing the significance of the difference between their correlations (see “Methods” for details). In general, our results here call into question the usefulness of using satellite-derived primary productivity and temperature to capture variation in export efficiency for the SO, at least in the AZ where NPP may be underestimated due to deep phytoplankton blooms^34^. Given that there are different relationships between e-ratio and environmental variables (e.g. NPP and SST), the region-specific parametrizations of e-ratio should be identified to reduce the uncertainty of the estimates in carbon export in the SO.

### Temperature dependence of export efficiency

As recently proposed by Britten et al.^14^, the weak correlation between temperature and export efficiency in the SO may be explained by the contrary response of temperature-driven respiration and particle ballast to export efficiency. The hypothesis is that the ballast-driven sinking of particles obscures the negative effect of temperature-dependent remineralization on e-ratio in the SO. Thus, when changes in diatom-related silica ballast are taken into account, the expected negative temperature-efficiency relationship is recovered (see Fig. 3D in Britten et al.^14^).

**Figure 3:**
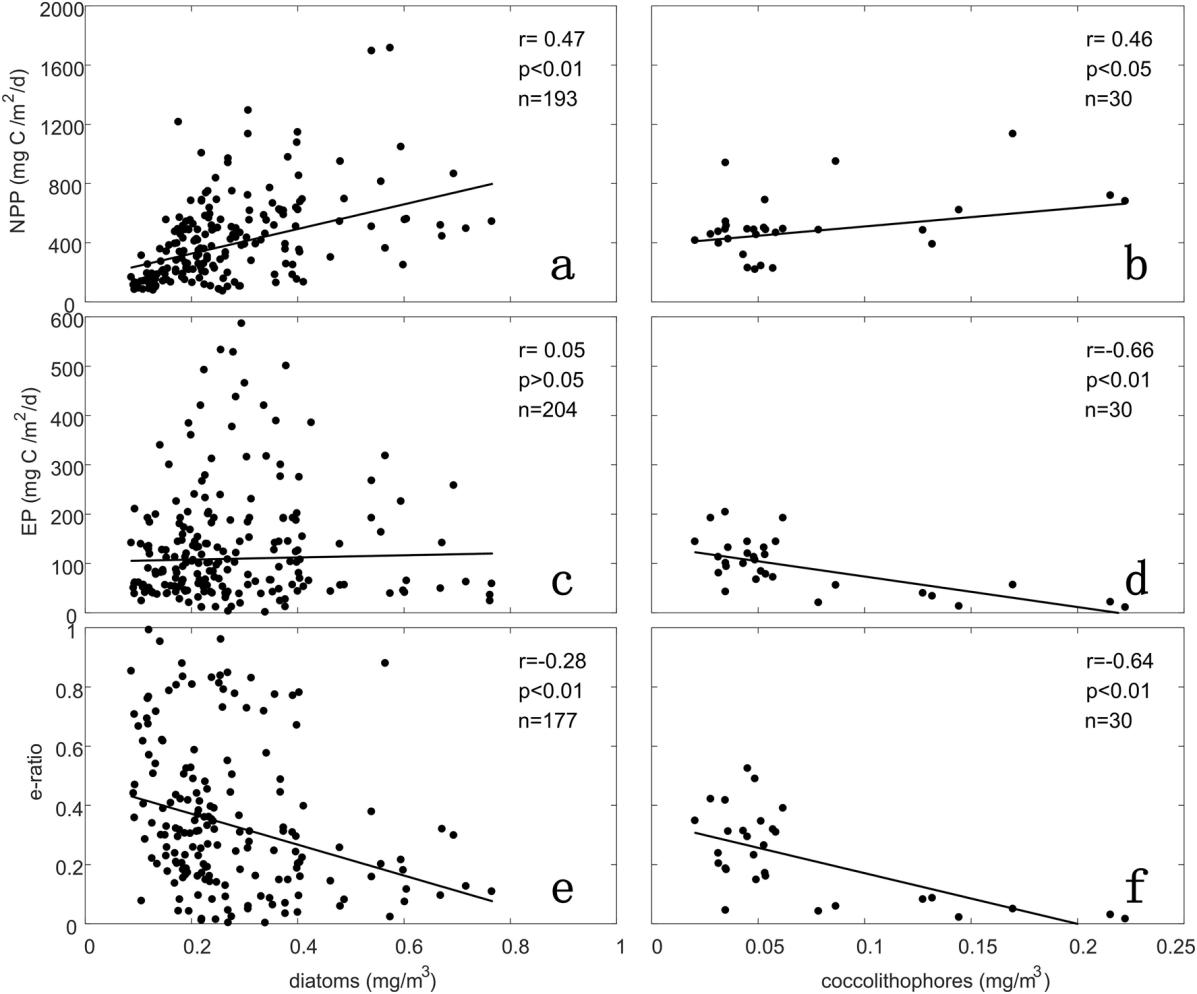
Relationships of diatom **(a, c, e)** and coccolithophore **(b, d, f)** concentrations with NPP **(a, b)**, EP **(c, d)**, and e-ratio **(e, f)**. The black line in each panel indicates the least square linear regression. Pearson’s correlation coefficients (*r*-value) and *p*-values are shown. Note that all e-ratio (EP/NPP) values are calculated using VGPM NPP.

As such, to examine whether a ballast-driven increase in the sinking speed of POC elevates carbon export efficiency, the concentrations of silicifying and calcifying phytoplankton were extracted from the NASA Ocean Biogeochemical Model (NOBM)^35^ outputs. Although there is still a significant challenge to model the phytoplankton composition and mineral ballasting was not accounted for in the NOBM, this model has been validated by both a publicly-available in situ dataset and remotely sensed estimates of phytoplankton group distributions^35–36^. It facilitates to describe the regional distributions of key phytoplankton groups (e.g., diatoms and coccolithophores) rather than the absolute accuracy of phytoplankton concentrations in the SO. We can see that the increase in satellite-derived NPP and the decrease in inferred e-ratio are associated with the NOBM predicted increase in diatom or coccolithophore concentration (Fig. 3a, b, e, f). These results suggest that the export efficiency of POC would not increase simply with the mineral ballast, and thus, in contrast to the “temperature-ballast hypothesis”^14^, it cannot explain the weak temperature dependence of e-ratio. Figure 3c, d show that blooms of coccolithophores even restrain POC export (*r* = − 0.66, *p* < 0.01), whereas the effect of diatom bloom on EP is uncertain (*r* = 0.05, *p* > 0.05). In contrast to our findings, Rosengard et al.^37^ found that the ^234^Th-based POC export was correlated with biogenic silica export, but not with PIC export. They hypothesized that these correlations are associated with the differences in size and lability of particles exported by the dominant phytoplankton communities. One major difference between our results and those by Rosengard et al.^37^ is the indicator of the ballast effect (mineral-associated phytoplankton concentrations versus mineral export fluxes). Although both studies do not deny the influence of ballast on the export of POC, it could not be completely separated from the roles of phytoplankton communities and associated food webs. Thus, the mineral ballast may not be parameterized adequately as a single mechanism enhancing surface carbon export. Instead, it should be integrated as part of the complex processes in the plankton community structure and function^13^.

Considering the unique marine ecosystem in the SO (i.e., strong seasonality, high grazing activity, diatom-dominated phytoplankton), the independence of export efficiency to low temperatures suggests that there are additional, and potentially more important processes, that have been overlooked besides mineral ballast. Laws et al.^7^ first discovered that e-ratio was insensitive to NPP and negatively correlated with temperature in steady-state systems, and that temperature alone explained 86% of the variance in e-ratio on a global scale. However, many factors including export lagging production and/or lateral advection would lead to a non-steady state condition^20,38,39^. Moreover, most stations in their study were located at low latitudes except for one station in the Ross Sea of Antarctica. Recently, a simple mechanistic model based on thermodynamic theory was proposed to estimate the e-ratio dependence on temperature^40^. It was suggested that the SO e-ratio has increased slightly due to cooling between 1982 to 2014^41^, which contrasts with our results reported here. The metabolic model in Cael et al.^41^ assumes that metabolic activity is based solely on temperature and does not include energy demand and substrate availability. From a bottom-up view, primary productivity in the high-latitude ocean is mainly limited by the availability of light and iron^42^. The weak temperature effect on NPP could be attributed to the physiological adaptation of phytoplankton (e.g., diatoms) to cold waters in the SO. Therefore, we hypothesize that the scarcity of observed temperature dependence on export efficiency in the SO is related to the role of temperature in phytoplankton photosynthesis.

### HPLE regime

Like temperature, NPP has been widely used as a parameter in evaluating e-ratio. The time lag between NPP and POC export accounts for a large percentage (up to 60%) of the variance in the relationship between instantaneous NPP and e-ratio^20^. However, our method mitigates this effect by using the averaged satellite-derived NPP rather than instantaneous measurements. There are three major pathways for the fate of organic carbon in the surface ocean: consumption by heterotrophic respiration, accumulation as non-sinking organic carbon (suspended particulate and dissolved organic carbon), and sinking POC. The last two components largely make up the net community production (NCP). Given the key role of heterotrophic respiration on the consumption of net primary produced organic matter, recent studies speculated that the inverse relationship between e-ratio and NPP can be explained by zooplankton abundance and bacterial activity^23,24^.

**Figure 4:**
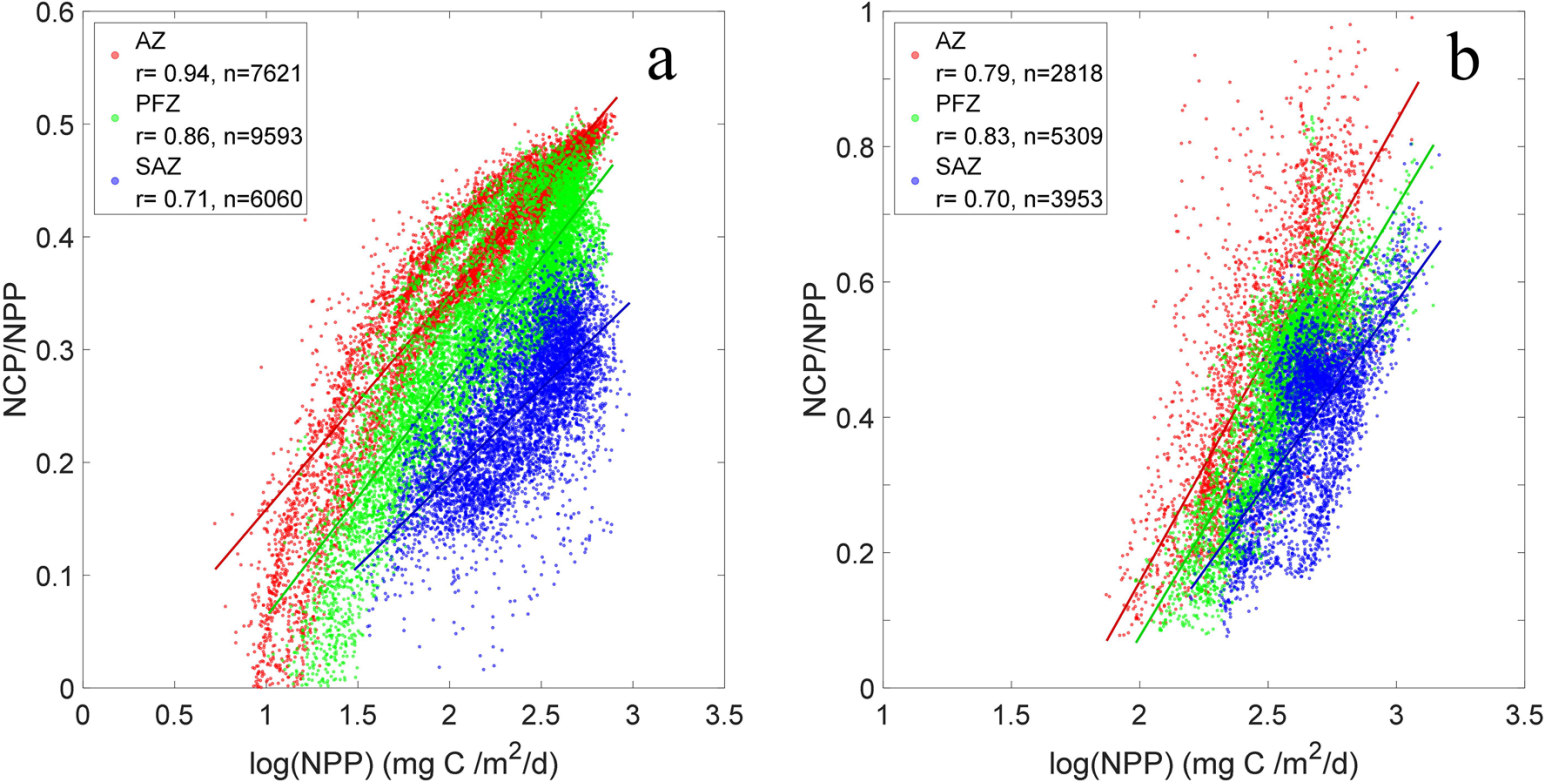
Depth-integrated (0–100 m) NCP/NPP as a function of NPP from the B-SOSE **(a)** and CESM **(b)** model outputs in each biome (red: AZ; green: PFZ; blue: SAZ). All correlations are above the 99% confidence level (*p* < 0.01).

To evaluate the impact of heterotrophic respiration on export efficiency, we use outputs from both the Biogeochemical Southern Ocean State Estimate (B-SOSE) model^43^ with data assimilation and the fully coupled Community Earth System Model (CESM)^44^. Figure 4 shows the response of NCP/NPP to changing NPP in different biomes as defined by Fay and McKinley^31^. Both models indicate that there are significant linear correlations between NCP/NPP and NPP in all biomes (*r* > 0.7, *p* < 0.01). Ignoring the lateral advection of organic carbon, the fraction consumed by heterotrophs should be balanced by the difference between NCP and NPP, thus inferring a decreasing ratio between heterotrophic respiration and NPP with increasing NPP, although heterotrophic respiration accounts for 50–90% of NPP and increases during phytoplankton blooms. The model results do not agree with those reported by Le Moigne et al.^23^, which suggested that intensive upper-ocean recycling could account for a large fraction of organic carbon loss and then cause HPLE.

Given that NCP, which is composed of sinking and suspended POC and also DOC, represents a larger portion of organic carbon originated in increased NPP (Fig. 4), we infer that the suspended fraction of organic carbon could be responsible for the HPLE regime to a large extent. The main reason for this speculation is that the POC export flux data that we compiled were measured based on either ^234^Th method or sediment traps, and both methods ignore the contributions of suspended organic matter subduction and active carbon transport induced by zooplankton vertical migration^39^. For example, by identifying the anomalies of physical (spiciness) and biological (oxygen and optical backscatter) variables based on a large biogeochemical-Argo database, Llort et al.^45^ found that the contribution of subduction events driven by eddies to EP ranged between 1 and 19%, but could rise to more than 100% for particularly strong events.

As stated earlier, there is no significant inverse relationship between NPP and e-ratio in the AZ (*r* = − 0.28, *p* > 0.05; Fig. 2d), and this could be caused by ballast materials (opal, lithogenic) and high fecal pellets with strong diel and seasonal grazing at the surface and excretion at depth by vertically migrating zooplankton in contrast to the situation in low-latitude regions. Specifically, the different effects of the two ballast minerals on POC export shown in Fig. 3c, d suggest that opal ballast during diatom blooms has the potential to introduce stronger POC export from the upper ocean than the ballast of sinking coccolithophore calcite. Cavan et al.^24^ observed that export efficiency in the Scotia Sea was closely (40%) related to zooplankton fecal pellets, which dominated the POC flux. Similarly, based on decades of krill density data, Belcher et al.^46^ reported that krill fecal pellet POC export accounted for 17–61% of the total seasonal carbon flux during the productive period in the marginal ice zone of the SO. Given the stronger seasonality of the plankton community in the AZ compared to that in the other regions of the SO, zooplankton could play a bigger role in mediating the transport of organic carbon in this region^25,47^. Moreover, the consumption and excretion by higher trophic levels (e.g., mesopelagic fish, whale) would exacerbate the active vertical transport and loss of the surface organic carbon^48^, which needs to be explored further in the regions with strong fish activities.

### Accumulation of non-sinking organic carbon

On global and annual scales, it is estimated that 20–25% of organic carbon is exported in the dissolved phase to below 100 m^49,50^, but just ~ 10% in the AZ and PFZ and ~ 20% in the SAZ below 74 m^51^. The downward flux of DOC at 100 m, determined by multiplying the vertical gradient of DOC by vertical diffusivity coefficient, was only 1–10% of the POC export flux in the Scotia Sea of the SO^23^. However, an inverse modeling study, constrained by hydrographic parameters, dissolved nutrients, oxygen, and carbon, suggested that annual DOC export flux amounts to 29% of NCP at 133 m^52^. Recently, results from several ocean models indicate that satellite-based estimates of POC export underestimate the seasonal amplitude of NCP by a factor of 2 or greater, implying that seasonal production of DOC contributes significantly to NCP^53^. These studies support our hypothesis that DOC accumulation and subsequent export via physical processes may be one of the potential reasons for the underlying HPLE phenomenon.

On the seasonal time scale, DOC could be an important component of NCP, of which the semi-labile DOC is resistant to a rapid decay in the euphotic zone but attenuates dramatically in the mesopelagic zone, and therefore largely contributes to carbon export over periods of several months to years^49^. Letscher et al.^54^ found that surface DOC in the eastern tropical South Pacific was resistant to microbial remineralization in the surface water for 9–14 days, but could be consumed by mesopelagic microbial communities. These findings imply that seasonal accumulation of organic carbon in dissolved forms causes a limitation of both heterotrophic respiration and POC export. Unfortunately, to the best of our knowledge, little field research has been conducted in the SO to directly evaluate the contribution of DOC to NCP or NPP.

**Figure 5:**
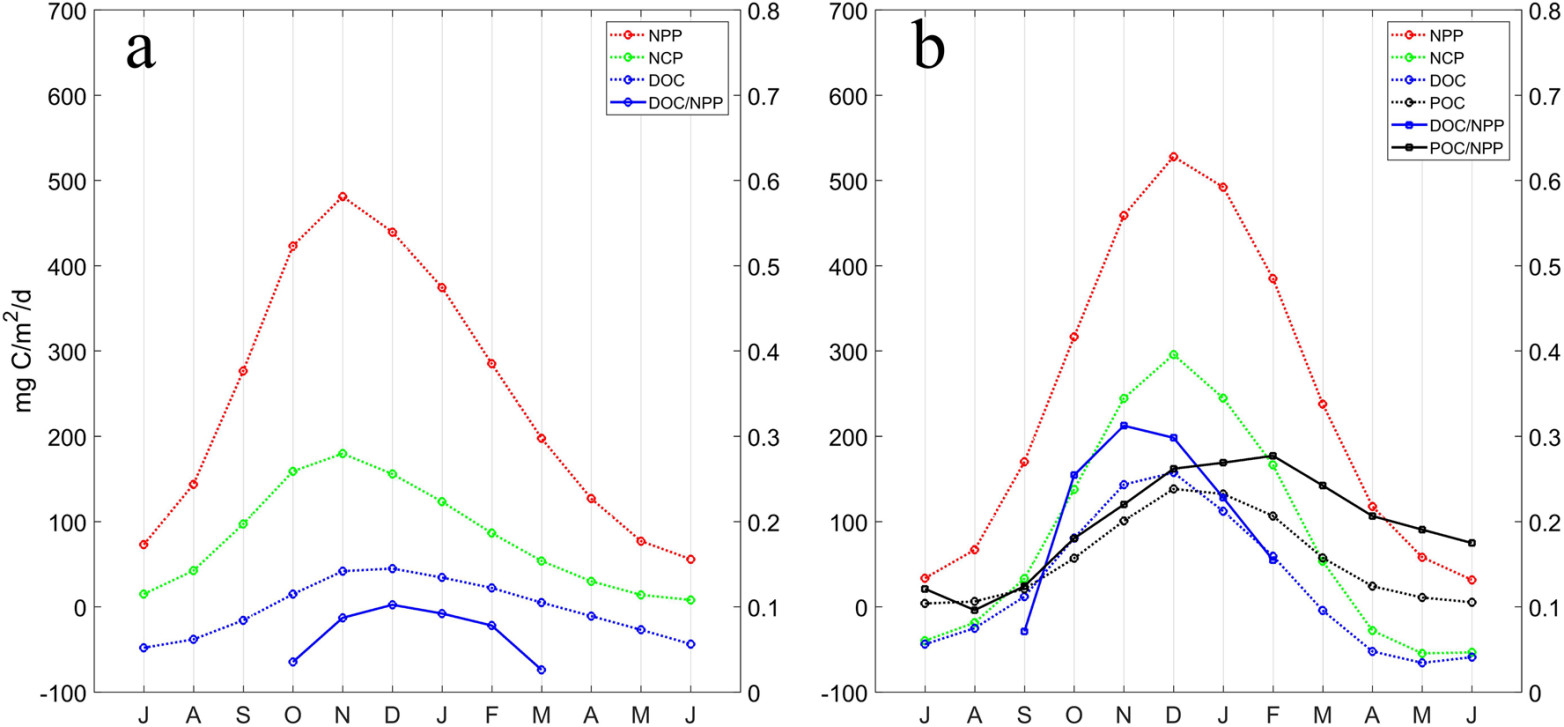
Monthly climatology of depth-integrated (0–100 m) NPP, NCP, DOC, POC (*left Y*-axis), and the ratio between DOC (POC) and NPP (*right Y*-axis) in the PFZ and SAZ extracted from the B-SOSE **(a)** and CESM **(b)** model outputs.

Figure 5 presents the monthly variations of climatological net DOC production, POC export, and their contributions to NPP in the PFZ and SAZ using outputs of B-SOSE and CESM models. The maximum contribution of DOC to NPP varies from 10 to 30% depending on model simulations, but both models show the increased contribution of DOC to NPP with increasing NPP, especially during spring blooms. The variation in DOC/NPP reflects the changes in both the quality and quantity of DOC released or consumed by ecosystem community^49^. The monthly climatology of DOC flux accumulates seasonally in surface waters and displays similar temporal variations to that of NCP and NPP (Fig. 5). These fluxes of DOC together with suspended particles are either remineralized later by heterotrophic organisms or exported through physical processes (e.g. subduction driven by eddies and mixed layer shallowing)^55^.

Since suspended POC is not a standard diagnostic in these biogeochemical models, we could not assess its contribution to NPP. Recently, Dall'Olmo et al.^56^ demonstrated that the contribution of seasonal variations in mixed layer depth, which could transport non-sinking particles below 100 m, to carbon export amounts to 100% in the areas of SO with deep winter mixed layers through satellite-based POC concentration and mixed-layer depth from Argo floats. By combining the Palmer Long Term Ecological Research program (LTER) data set with autonomous profiling float data, Stukel and Ducklow^57^ estimated that passive export of suspended POC and DOC by vertical mixing occupied 25–50% and 3–6%, respectively, of the total exported organic carbon. In fact, more than 90% of the POC pool is retained in the surface ocean, and does not immediately sink out of the upper water^58,59^. POC accumulation in the upper-ocean waters could reach up to ~ 50% of NCP in the Ross Sea^60^, the Amundsen Sea^61^, and the region around the Crozet Islands^62^. Thus, in addition to DOC accumulation, suspended POC should also be an important component of organic carbon budget^39^, and could be another possible factor driving the HPLE regime in the SO. In the highly productive regions (e.g. marginal ice zone), phytoplankton growth is supported by iron supply from melting sea ice and strong stratification of the water column with light availability^63^. This relatively stable physical environment would favor a temporary accumulation of non-sinking organic carbon in the upper 100 m, which results in a decrease in e-ratio.

More recently, HPLE regimes have also been observed in other regions of the global ocean, such as the North Atlantic^64^, the northern Gulf of Mexico^65^, the Arabian Sea and Indian Ocean^66^, and the California Current ecosystem^67^. Despite the dominance of temperature in carbon export at global scale^7,41^, it is reasonable to surmise that other processes, such as accumulations of DOC and POC in the surface, can largely determine the efficiency of carbon export on the regional and seasonal scale. More field observations and validations are required to construct a complete picture of the effects of DOC and POC accumulations on carbon export efficiency.

## Conclusions

Based on the analysis of an in situ dataset of EP compiled from scientific literature and environmental parameters from satellite products, we have demonstrated that carbon export efficiency is independent of temperature when the temperature is below 6 °C and that HPLE regimes are widely distributed in the SO except for the AZ. Our analysis of the ocean biogeochemical model outputs suggests that ballast minerals play less important roles in controlling export efficiency than ecosystem structure which may contribute significantly to the relative independence of export efficiency on temperature. HPLE regimes could be largely explained by the seasonal accumulation of organic carbon in the surface ocean**,** in contrast to what has been previously reported. These analyses provide alternative explanations of the observed patterns between export efficiency and potential drivers. In a warming SO, when more iron and light would become available for phytoplankton growth, NPP might increase and phytoplankton community may shift toward small size^63,68^, thus the carbon export efficiency would tend to decrease with more organic carbon accumulated and remineralized in the upper ocean; however, how carbon export production will change remains unknown. More field observations are needed to improve our understanding of the BCP in the SO and its potential climatic and ecological effects under climate change.

## Data and methods

### In situ EP

In this study, we compiled all the available field measurements of EP in the SO based on the ^234^Th-^238^U disequilibrium method and free-drifting sediment traps. Field measurements based on other approaches such as geochemical mass balance (nutrient, oxygen, or carbon) and incubation experiments (i.e., nitrate-based “new” production) were not used here, because these methods include both particulate and dissolved organic carbon exported out of the surface productive zone. We only chose observations at the depth of 100 ± 10 m and excluded repeated measurements. Although different choices of depth horizon could potentially lead to a discrepancy in export flux estimate, previous studies indicated that the choice of the depth horizon has little effect on the evaluation of EP and e-ratio in the SO at the euphotic depth or 100 m^69^. Also, observations in shallow waters (< 300 m) were eliminated to rule out the effect of sediment resuspension. The POC export flux in the period of 1997–2013 was mainly measured between October and April when there was sufficient solar radiation. In total, 218 of the 231 stations were analyzed with the ^234^Th-based method at the depth of 100 m; the remaining 13 stations were sampled by drifting sediment traps (Fig. 1). Limited by the remoteness and hostile environment, the sampling stations in the SO are not evenly distributed spatially and temporally over the past decades. Only a few observations were conducted in the Pacific sector and coastal regions around Antarctica and almost no data were available in winter. The observations are mainly condensed in the Southern Atlantic and near islands in the Indian sector. Still, the observational coverage extends from open-ocean high-nutrient low-chlorophyll (HNLC) waters to natural iron fertilization regions (e.g. downstream of South Georgia, Crozet, the Kerguelen Plateau) and across the seasonal sea ice zones. Several latitudinal sections across different fronts in this dataset could reflect the biogeochemical characteristics associated with carbon export among circumpolar zonal systems. Across the 231 stations, the EP values range from 1.2 to 586.2 mg C m^−2^ day^−1^, measured near the Subtropical Front and south of the Ross Sea, respectively, with the mean value of 130.5 mg C m^−2^ day^−1^.

Accounting for the uncertainty of the EP methods, there have been disparities of flux estimates between methods^70^. A study by Buesseler et al.^71^ found that the EP estimated from the drifting trap was nearly doubled to that observed by the ^234^Th approach on the shelf of the West Antarctic Peninsula. Given that the majority of the EP assembled were measured by the isotopic method (218/231) and only 13 stations are distributed across different regions and months, discrepancies in our estimate related to differences in observational methods are assumed to be negligible.

### Satellite-derived NPP and SST

Corresponding to the EP measurements described above, there were very limited measurements of in situ ^14^C-NPP. In contrast to the EP measurements that represent the average over several weeks, NPP in the euphotic zone was measured instantaneously during incubation, which lasted only several hours. The temporal difference between these two measurements could result in pronounced decoupling^38,72,73^. To overcome these problems, we used satellite-derived NPP products with a 1/6° spatial resolution and 8-day temporal resolution for the period 1997–2013, obtained from the Ocean Productivity website at Oregon State University (https://www.science.oregonstate.edu/ocean.productivity/). Three different NPP products were used based on different algorithms: chlorophyll-based vertically generalized productivity model (VGPM; ref.^74^), the Eppley-VGPM (Eppley; ref.^75^), and carbon-based productivity model (CbPM; ref.^76^). Overall, the differences between these models reflect differences in sensitivity of photosynthetic rate to temperature and to different indicators of phytoplankton biomass.

The daily mean Optimum Interpolation Sea Surface Temperature (OISST) dataset, with a horizontal resolution of 0.25°, was obtained online ( https://www.esrl.noaa.gov/psd/data/gridded/data.noaa.oisst.v2.html).

### Model outputs

The NOBM is a global three-dimensional biogeochemical model coupled with the Poseidon ocean general circulation model^35–36^, which spans from 84° S to 72° N at the resolution of 0.67° (lat) × 1.25° (lon). The biogeochemical component of the model distinguishes four phytoplankton taxonomic types (diatoms, chlorophytes, cyanobacteria, and coccolithophores), of which the relative abundances were positively correlated with in situ data, to represent the global patterns of marine phytoplankton diversity. It also includes four nutrients (nitrate, ammonium, silicate, and iron), three detrital pools (nitrate/carbon, silicate, and iron) and a single herbivore group. The NOBM products of daily diatom and coccolithophore concentrations from 1998 to 2015, were obtained from the Giovanni website (https://giovanni.gsfc.nasa.gov/giovanni).

Monthly NCP and NPP from 2008 to 2012 were estimated using the state-of-the-art B-SOSE model, configured with 1/3° horizontal resolution and 52 vertical levels^43^. The model was optimized to fit several biogeochemical Argo and in situ products. Since the net DOC production estimate is not a standard output of the B-SOSE model, we estimated the implicit net DOC production by calculating monthly differences in DOC concentration based on dissolved organic phosphorus (DOP) standard product and using an appropriate ratio of ^225^C:^19^N:^1^P (ref.^50^).

Outputs of the CESM Medium Ensemble from 2006 to 2010^44^, including NPP, NCP, POC export, and net DOC production, were also analyzed to assess the variability in the ratio of different components of organic carbon to NPP. The CESM model has a 1° horizontal resolution and 60 vertical levels, and has been used extensively for studying the SO^53,77,78^. Considering the small contribution of organic carbon as live plankton assemblages to NCP^53^, we assumed that NCP describes the net amount of organic carbon in the forms of POC and DOC produced by the plankton community. For the outputs of each model, we calculated the depth-integrated (0–100 m) and monthly climatological values, and then resampled these results at 3° resolution. For consistency, we used the NPP from models instead of satellite products when analyzing the outputs of the two models.

### Matchups with in situ EP

To find the concurrent and co-located satellite-derived NPP for each in situ observation of EP, 3 × 3 pixel boxes were used; and the derived NPP was then averaged over 24 days (the approximate half-life of ^234^Th) and 8 days (the duration of drifting trap sampling) prior to each EP measurement using the ^234^Th method and sediment trap, respectively. Although the 8-day averaged NPP values matched with sediment trap data are not time integrals due to cloud cover, their effects can be ignored because of the very small sample amounts (13/231). We assumed that doing so could remove the time lag effects between POC production and export to a large extent^73^. SST was extracted as the median of a 3 × 3 pixel box centered at each EP location on the same day, but the nearest grid point was used for matchups from NOBM outputs.

### Statistical analysis

NPP products were log-transformed and we excluded the single outlier of diatom concentration of 1.18 mg m^−3^ extracted from NOBM model before the analysis. To estimate the relation between two parameters (e.g. e-ratio, SST, and NPP; phytoplankton concentrations and carbon flux), we applied robust linear regression to reduce the effects of possible outliers. It should be noted that the linear regression we used does not mean that the underlying relationship is linear, but rather to explore the potential patterns in covariation between variables. Additionally, the Pearson pairwise correlations between the variables were examined and the statistical significance was indicated by p values < 0.05. All statistical analyses were performed using MATLAB R2017a.

While the methodological uncertainty of the satellite-based NPP estimates is not particularly well defined in the SO, we tried to validate these NPP products with in situ NPP. The nearest pixel values around each sampling station were extracted from the satellite-derived NPP products and compared with in situ measurements. The correlation analysis and the scatter plot reveal that the satellite-based NPP estimates generally agree with in situ measurements, with the exception that the CbPM underestimates largely NPP values (Supplementary Fig. S3) mainly during austral spring (October and November, not shown). This underestimation by the CbPM likely results from non-resolving vertical variations in phytoplankton chlorophyll:carbon ratio in the SO. The effects of uncertainties in NPP products on our e-ratio estimates were further evaluated below.

The e-ratio values greater than 1 (namely, EP > NPP) were excluded in this study, reducing the underestimation effects of NPP products to some extent. All the logarithm of e-ratio values derived from in situ EP and different satellite-derived NPP exhibited a normal distribution, which was justified by the one-sample Kolmogorov–Smirnov test at the 1% significance level. To assess the uncertainties in e-ratio induced by NPP products, we compared e-ratio values using in situ and satellite-based NPP without considering the time lag between primary and export production. Results show that these e-ratio values are generally in good agreement, with Pearson’s *r*-values ranging from 0.33 to 0.43 (*p* < 0.01, Supplementary Fig. S4). We then performed a Fisher’s *r* to *z* transformation to check the sensitivity of the relationship between e-ratio and NPP to the choice of NPP products. The negative relationship of e-ratio to NPP for all data points is broadly similar for both in situ and satellite-based NPP (Supplementary Fig. S5) and there is no significant difference between the correlation coefficients (Fisher’s *z* test, *p* = 0.8, 0.25, 0.72 for VGPM, Eppley-VGPM, CbPM versus in situ, respectively). These indicate that our e-ratio estimates and their relationships with environmental factors are less sensitive to the satellite-based NPP products as also suggested by Figs. 2 and S1-S2.

## Supplementary information

Supplementary Information 1.

Supplementary Information 2.

## Data Availability

Published particulate organic carbon export flux data from in situ measurements during 1997–2013 were compiled (Fig. 1, and supplementary materials). The B-SOSE model outputs used here are freely available at https://sose.ucsd.edu/BSOSE_iter105_solution.html. The CESM Medium Ensemble outputs are available at the Earth System Grid (https://www.earthsystemgrid.org/).
