# Supplementary material for: Southern Ocean carbon export efficiency in relation to temperature and primary productivity
Source: Sci Rep. 2020 Aug 10;10:13494. doi: 10.1038/s41598-020-70417-z (PMC7417578; doi:10.1038/s41598-020-70417-z)
Supplement: Supplementary file 1 — Supplementary Information 1. [file 41598_2020_70417_MOESM1_ESM.pdf]

Supplementary Information for

Southern Ocean carbon export efficiency in relation to temperature and primary productivity

Gaojing Fan[1,2,3], Zhengbing Han[2*], Wentao Ma[3*], Shuangling Chen[3], Fei Chai[3,4], Matthew R. Mazloff[5], Jianming Pan[2], Haisheng Zhang[1,2]

[1] College of Marine Science and Technology, China University of Geosciences, Wuhan, 430074, China

[2] Key Laboratory of Marine Ecosystem Dynamics, Second Institute of Oceanography, Ministry of Natural Resources, Hangzhou, 310012, China

[3] State Key Laboratory of Satellite Ocean Environment Dynamics, Second Institute of Oceanography, Ministry of Natural Resources, Hangzhou, 310012, China

[4] School of Marine Sciences, University of Maine, Orono, ME 04469, USA

[5] Scripps Institution of Oceanography, University of California San Diego, La Jolla, CA 92093, USA

*Corresponding authors: Zhengbing Han (hzbing@sio.org.cn)

Wentao Ma (wtma@sio.org.cn)

Contents of this file

Figures S1-S5,

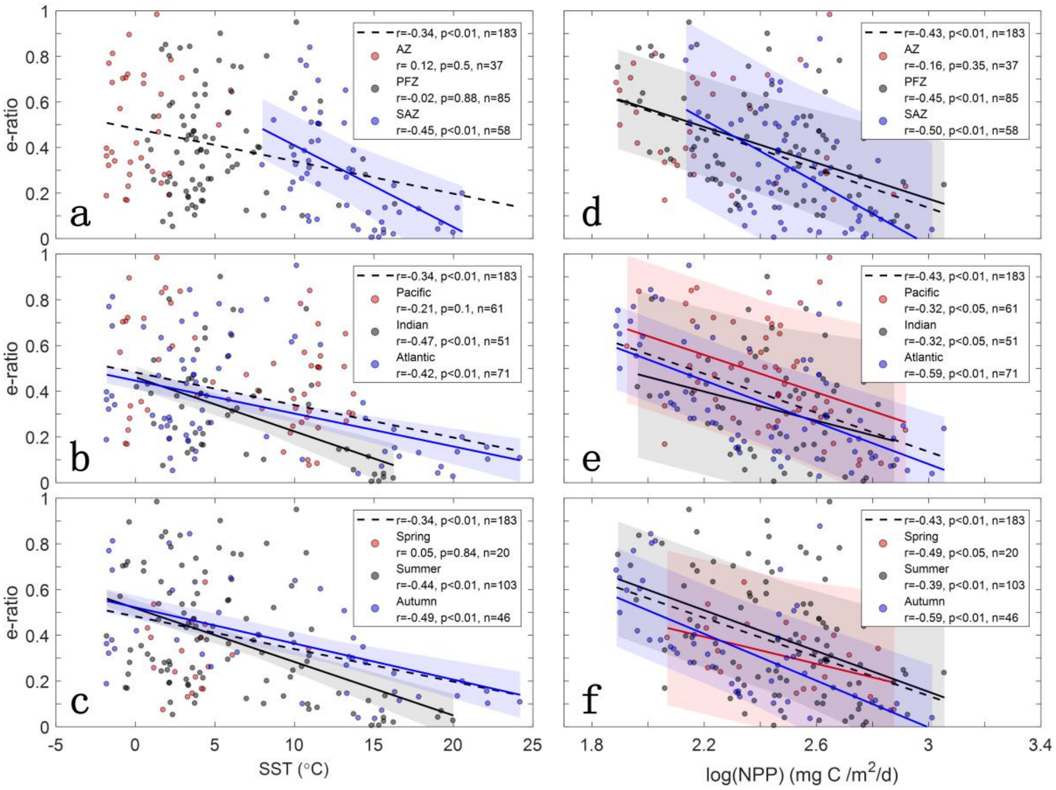

Figure S1. Same as Fig. 2 but for the satellite-based NPP using Eppley-VGPM.

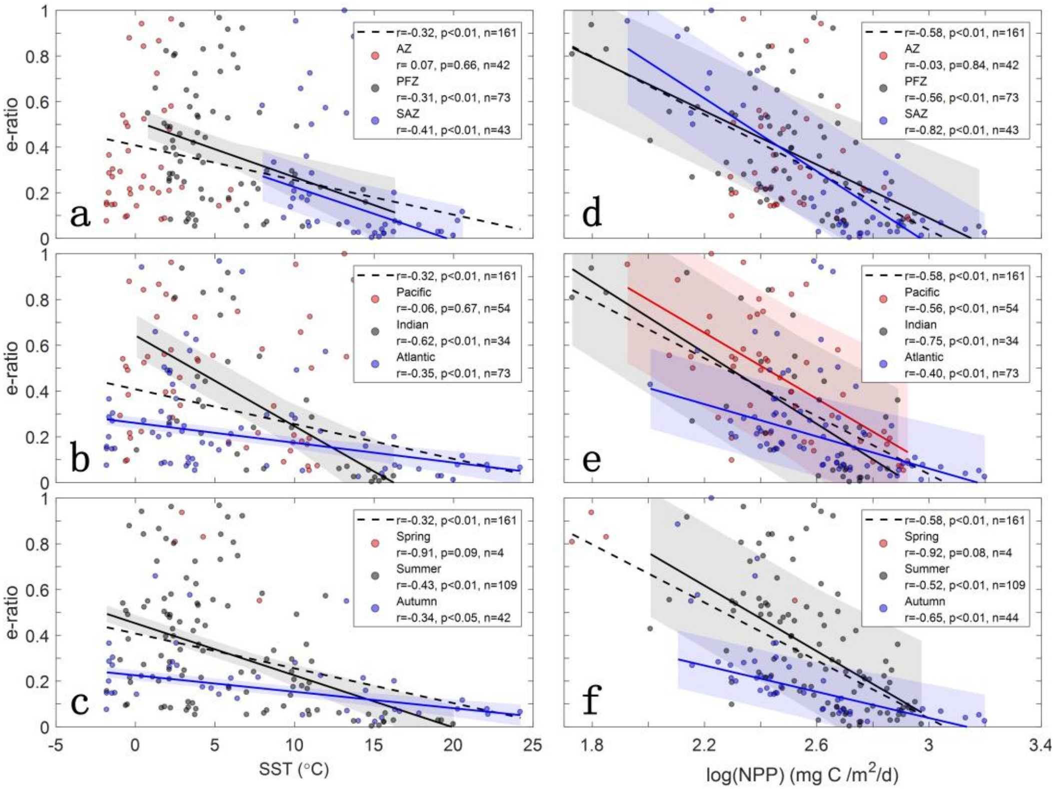

Figure S2. Same as Fig. 2 but for the satellite-based NPP using CbPM.

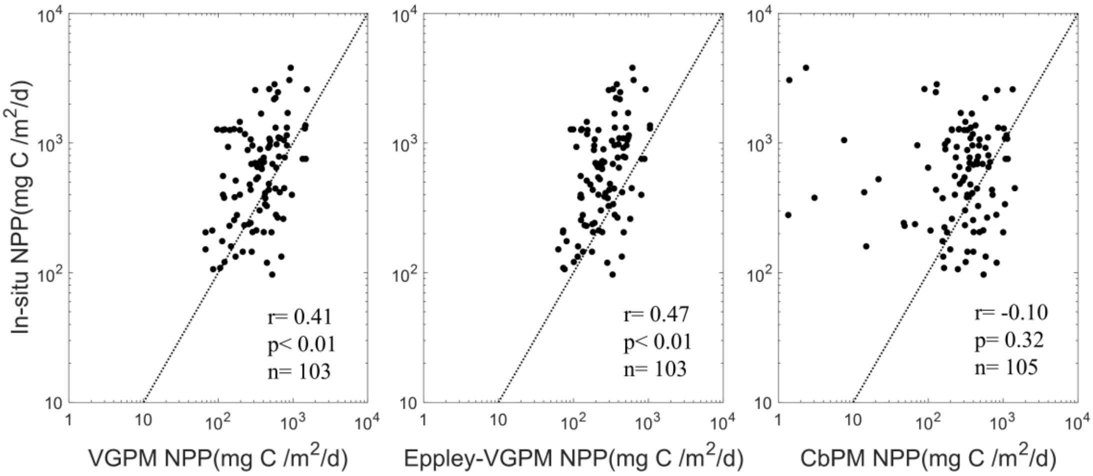

Figure S3. Comparison of *in situ* and satellite-based NPP on a log scale. Note that only *in situ* NPP synchronously measured with EP were used, not all NPP observations in the Southern Ocean. The dotted line is the 1:1 line in each panel. Pearson's correlation coefficients (*r*-value) and *p*-values are shown.

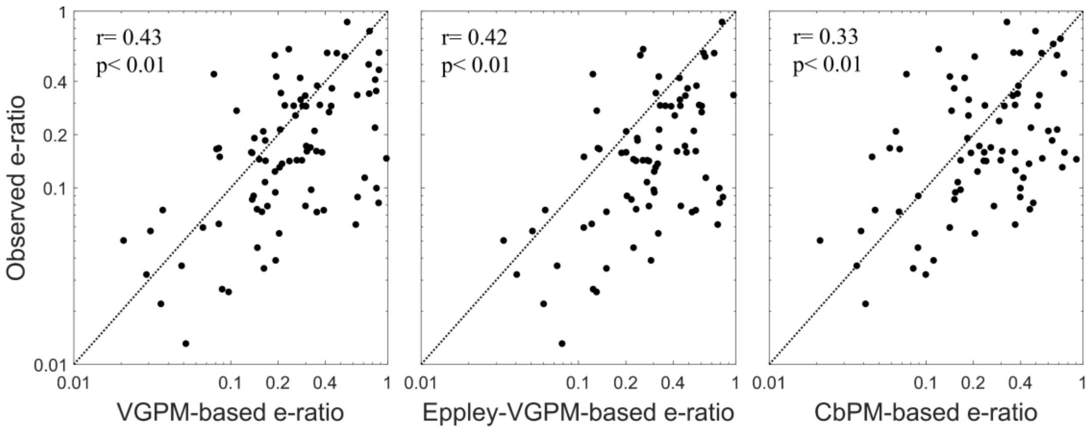

Figure S4. Comparison of the e-ratio estimates using *in situ* and satellite-based NPP from Fig. S3 on a log scale, without considering the time lag between EP and NPP when calculating e-ratio. The dotted line is the 1:1 line in each panel. Pearson's correlation coefficients (*r*-value) and *p*-values are shown.

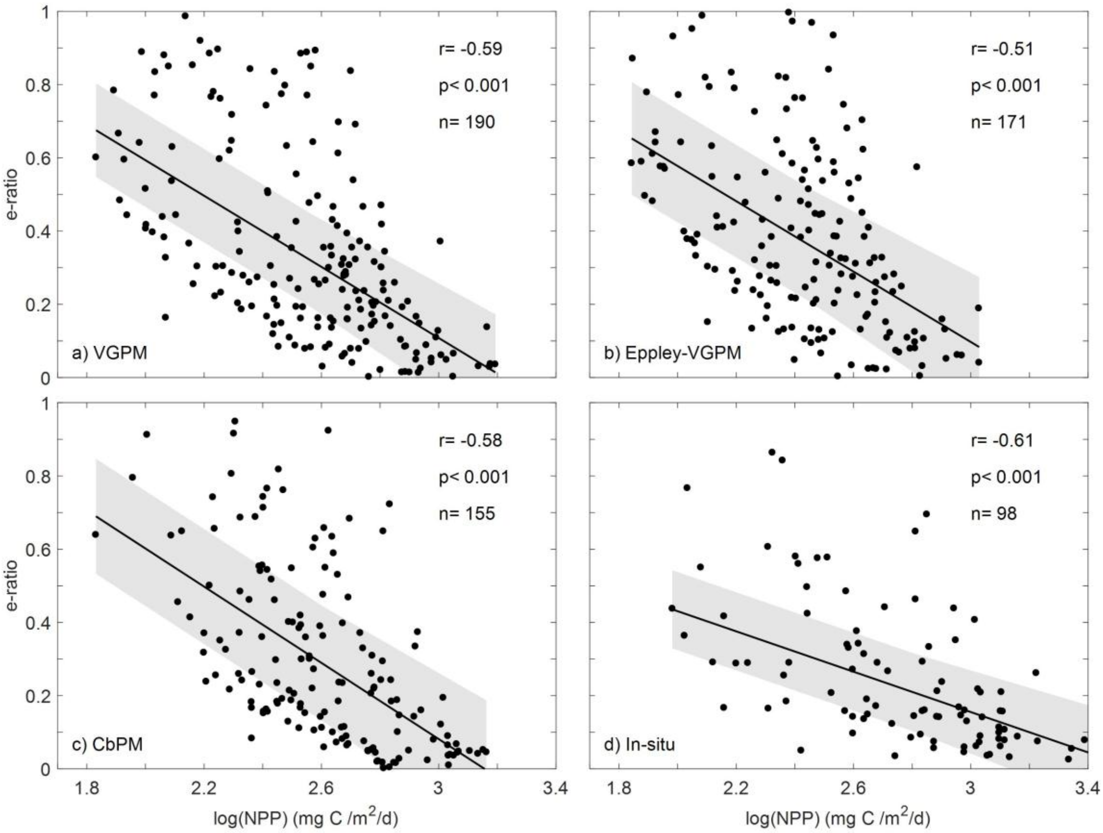

Figure S5. Comparison of the relationships between e-ratio and NPP from the satellite-based VGPM (a), Eppley-VGPM (b), CbPM (c), and *in situ* observations (d), without considering the time lag between EP and NPP when calculating e-ratio. The Fisher's r to z transformation was used to test the significance of the difference between the correlation coefficients (see Methods). The shaded areas represent the standard error around the fitted lines. Pearson's correlation coefficients (*r*-value) and *p*-values are shown.
